# Perceived behavioral control as a potential precursor of walking three times a week: Patient's perspectives

**DOI:** 10.1371/journal.pone.0192915

**Published:** 2018-02-16

**Authors:** Peter Busse, J. Jaime Miranda

**Affiliations:** 1 Universidad de Lima, Instituto de Investigación Científica, Lima, Peru; 2 Instituto de Estudios Peruanos, Lima, Peru; 3 CRONICAS Center of Excellence in Chronic Diseases, Universidad Peruana Cayetano Heredia, Lima, Peru; 4 Department of Medicine, School of Medicine, Universidad Peruana Cayetano Heredia, Lima, Peru; UNITED STATES

## Abstract

**Background:**

Behavior change theories can identify people’s main motivations to engage in recommended health practices and thus provide better tools to design interventions, particularly human centered design interventions.

**Objectives:**

This study had two objectives: (a) to identify salient beliefs about walking three times a week for 30 minutes nonstop among patients with hypertension in a low-resource setting and, (b) to measure the relationships among intentions, attitudes, perceived social pressure and perceived behavioral control about this behavior.

**Methods:**

Face-to-face interviews with 34 people living with hypertension were conducted in September-October 2011 in Lima, Peru, and data analysis was performed in 2015. The Reasoned Action Approach was used to study the people’s decisions to walk. We elicited people’s salient beliefs and measured the theoretical constructs associated with this behavior.

**Results:**

Results pointed at salient key behavioral, normative and control beliefs. In particular, perceived behavioral control appeared as an important determinant of walking and a small set of control beliefs were identified as potential targets of health communication campaigns, including *(not) having someone to walk with*, *having work or responsibilities*, or *having no time*.

**Conclusions:**

This theory-based study with a focus on end-users provides elements to inform the design of an intervention that would motivate people living with hypertension to walk on a regular basis in low-resource settings.

## Background

To increase the likelihood of success, health communication interventions tend to rely on behavior change theories in order to identify people’s main motivations to engage in recommended health practices. Several behavioral theories are available [1–7] and reviews stress the argument that program practitioners should rely on these theories to design successful preventive or lifestyle interventions [5, 8, 9]. Central to behavior change theories is the claim that health interventions impact behavior through a mechanism of influence: health messages—or any informational intervention for that matter—first, influence peoples’ beliefs, and, subsequently, these beliefs influence attitudes, self-efficacy or intentions, which in turn influence behavior [6]. These theories can, in turn, provide better tools to design interventions, particularly human centered design interventions.

This study applied one major behavior change theory, namely the Reasoned Action Approach [4, 6, 7], first, to identify salient beliefs about walking three times a week for 30 minutes nonstop among patients with hypertension and, second, to measure the relationships among intentions, attitudes, perceived social pressure and perceived behavioral control about this behavior in this population. Overall, as a risk factor for cardiovascular diseases, which are responsible for almost half the deaths resulting from chronic diseases in Latin America and the Caribbean [10], hypertension constitutes a problem relevant to public health interventions.

### Hypertension

Hypertension is a health condition affecting 26% of people worldwide [11]. Although its consequences include an increased risk for heart diseases or stroke [12], research points at unhealthy habits or the co-presence of other diseases as major risk factors [13]. Three main groups among individuals with the condition can be identified: (i) those that have the condition but do not know they have it, (ii) those who have the condition, know that they have it but do not control it, and (iii) those that have the condition, know that they have it and control it [14, 15]. Any health intervention aiming to improve the management of hypertension can target these groups independently, or collectively, and may address any of its determinants, for example physical inactivity [15].

A systematic review of randomized controlled trials has shown that effective interventions—that is, those causing decreases in measures of blood pressure through increased walking among people with hypertension—exhibited three characteristics: they were implemented over long periods of time (average of 19 weeks), included large samples of individuals and, for the most part, promoted intense walking (e.g., maximum heart rate greater than 80%) among their participants [16]. Thus, interventions addressing the management of hypertension can encourage individuals to walk on a regular basis; but doing so is not an easy endeavor.

### The Reasoned Action Approach

**Fig 1:**
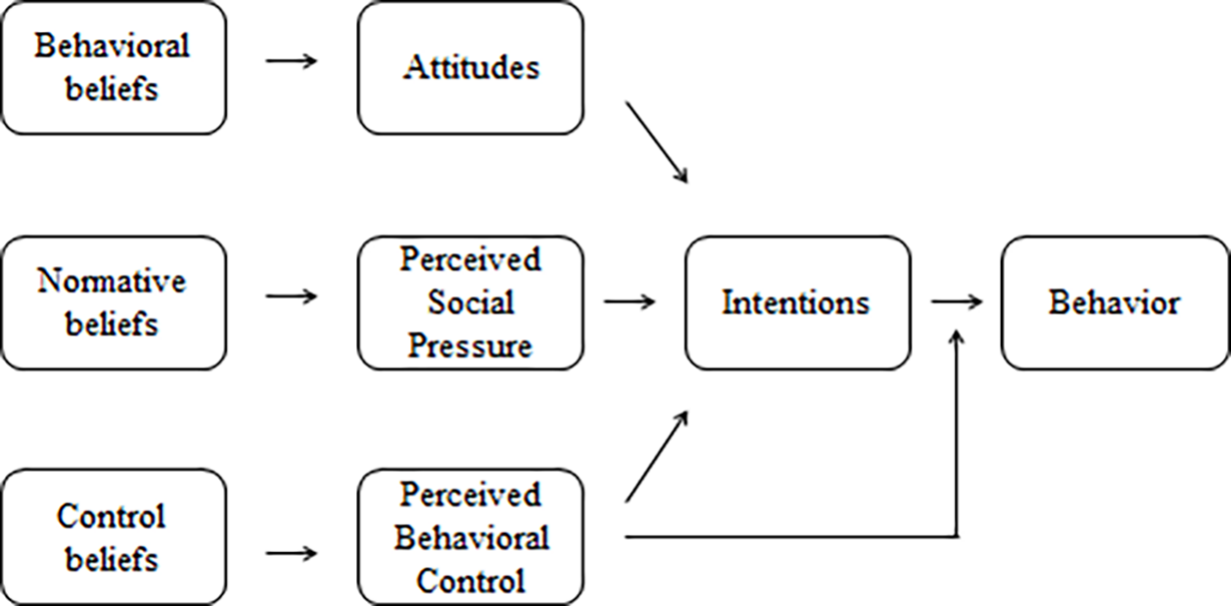
Reasoned Action Approach model. Source: Fishbein and Ajzen (4).

The theories underlying the Reasoned Action Approach [6] have been largely employed to examine the predictors of people’s behaviors as well as to design health interventions [9, 17]. For example, the model has been employed to design interventions to motivate safe sex practices, increase physical activity or decrease the consumption of sugary drinks in U.S. households [5, 18, 19]. Fig 1 shows the outline of the Reasoned Action Approach [4].

The Reasoned Action Approach postulates that any behavior can be predicted by people’s intentions to engage in the behavior [1]. According to the theory, people’s intentions are formed on the basis of three cognitive constructs: attitudes, perceived social pressure and perceived behavioral control [4]. The theory conceptualizes perceived social pressure as a combination of injunctive norms, or the perceptions of what ought to be done in a given situation, and descriptive norms, or the perceptions of what is done in that situation [4, 20].

Moreover, the theory proposes that each cognitive construct is formed on the basis of a set of beliefs [1]. Thus, people’s beliefs about the potential outcomes of a given behavior, or behavioral beliefs, form their attitudes towards the behavior; similarly, the beliefs about the social referents who approve or disapprove of their behavior form their injunctive norms; the beliefs about the social referents who engage or do not engage in a given behavior form their descriptive norms; and, finally, the beliefs about the facilitators and barriers to enact a given behavior form people’s perceived behavioral control [4]. For example, the beliefs that *walking would improve my health* and *walking would give me a chance to meet other people* would form an overall attitude towards walking; the beliefs that *my spouse approves of my walking* and *my friend also walks frequently* would form an overall perception of norm towards walking; and the belief that *having free time will help me walk regularly* would be the main contributor for an overall perception of control over a behavior. Thus, all these beliefs would be the basis for why people walk on a regular basis.

But, according to the theory, not every belief has a role in people’s decisions to engage in the behavior: only those beliefs that are salient in people’s minds are relevant—that is, only those that are readily accessible in memory are used to form attitudes, perceived social pressure and perceived behavioral control [4]. Following on the above example, and unlike the behavioral beliefs already indicated, it is possible that a third belief, such as *walking would make me feel connected with nature*, may not be salient in people’s minds when they form their attitudes towards walking on a regular basis. Overall, one advantage of the Reasoned Action Approach is that it provides a clear methodology for identifying those beliefs in a population of interest [4].

The objective of this study was twofold: to identify salient behavioral, normative and control beliefs about walking three times a week for 30 minutes nonstop among a sample of individuals with hypertension in Lima, Peru, and to measure the extent to which these individuals formed intentions based on attitudes, perceived social pressure and perceived behavioral control in regards to this behavior. Walking three times a week has been identified as a relevant behavioral target associated with positive health outcomes [21]. We focused on “30 minutes nonstop,” because we wanted to explore the possibility that patients could protect at least 30 minutes of their time to engage in this form of physical activity. Also, defining the frequency of the behavior as “three times a week” would help identify beliefs that are relevant to this frequency, but not to a lower frequency; for example, walking “once a week” could be perceived as achievable by a patient, but “three times a week” may not [4].

## Methods

### Participants and setting

Patients of two health centers—a national public hospital and a private clinic—in Lima, Peru, were interviewed face-to-face between September and October 2011 [15]. All participants were 18 years old or older, and only individuals who were diagnosed with hypertension by an attending physician, at any given time before the dates of data collection, were approached and interviewed by a trained research assistant. Individuals could have controlled or uncontrolled hypertension at the moment of the interview (values above 140 for systolic blood pressure or above 90 for diastolic blood pressured were regarded as uncontrolled hypertension).

### Measures

A module of the interview guide [15] was used to measure the constructs of the Reasoned Action Approach in regards to walking three times a week for 30 minutes nonstop [4]. The development of the instrument followed recommendations of Fishbein and Ajzen [4] to capture these constructs and, in order to assure comprehension of the items, the questionnaire was pre-tested with a sample of individuals living with hypertension, who were not part of the study’s sample. Thus, one subsection was devoted to the elicitation of the beliefs about walking three times a week for 30 minutes nonstop, including behavioral, normative and perceived behavioral control beliefs; and another subsection was used for measuring the theoretical constructs including attitudes, perceived social pressure, perceived behavioral control and intentions in regards to walking three times a week for 30 minutes nonstop. A single item measured whether participants engaged on this specific health behavior or not.

### Elicitation of salient beliefs

Following Fishbein and Ajzen [4], behavioral beliefs were elicited using two questionnaire items: *Tell me the advantages*, *the good or best things*, *of your walking three times a week for 30 minutes nonstop* and *Tell me the disadvantages*, *the bad or worst things*, *of your walking three times a week for 30 minutes nonstop*. Injunctive normative beliefs were elicited using the following two items: *Who are the most important people in your life who approve of your walking three times a week for 30 minutes nonstop*? *For example*, *your parents*, *siblings*, *partner*, *friends*, *etc*. and *Who are the most important people in your life who disapprove of your walking three times a week for 30 minutes nonstop*? *For example*, *your parents*, *siblings*, *partner*, *friends*, *etc*. Descriptive normative beliefs were elicited using the following two items: *Who are the people you know*, *or know of*, *who walk three times a week for 30 minutes nonstop*? *For example*, *your parents*, *siblings*, *partner*, *friends*, *etc*. and *Who are the people you know*, *or know of*, *who do not walk three times a week for 30 minutes nonstop*? *For example*, *your parents*, *siblings*, *partner*, *friends*, *etc*. Finally, perceived behavioral control beliefs were elicited using two items: *What makes easy*, *or what helps*, *your walking three times a week for 30 minutes nonstop*? *For example*, *a thing or a place*, *etc*. and *What makes difficult*, *or what barriers are there to*, *your walking three times a week for 30 minutes nonstop*? *For example*, *a thing or a place*, *etc*.

### The theoretical constructs

Following Fishbein and Ajzen [4], attitudes were measured by averaging eight bipolar semantic differential items from the stem *My walking three times a week for 30 minutes nonstop is…bad-good*, *foolish-wise*, *unnecessary-necessary*, *unimportant-important*, *not-enjoyable-enjoyable*, *unpleasant-pleasant*, *uncomfortable-comfortable* and *useless-useful*. Each item was scored on a 4-point scale with higher scores indicating more positive attitudes.

Similarly, perceived social pressure was measured by averaging both injunctive and descriptive norms. Injunctive norms were measured with three items, each with 4-response categories ranging from *completely agree* to *completely disagree*: *Most people important to me would approve of my walking three times a week for 30 minutes nonstop*, *Most people important to me think I should walk three times a week for 30 minutes nonstop*, *Most people important to me would like me to walk three times a week for 30 minutes nonstop*. Responses were recoded so that higher scores indicated more positive injunctive norms. Descriptive norms were measured with two items, each with 4-response categories ranging from *completely agree* to *completely disagree*: *Most people similar to me walk three times a week for 30 minutes nonstop* and *Most people similar to me will walk three times a week for 30 minutes nonstop*. Responses were recoded so that higher scores indicated more positive descriptive norms.

Perceived behavioral control was measured by averaging three items scored with 4-response categories ranging from completely agree to completely disagree: *Walking three times a week for 30 minutes nonstop will depend on me*, *Walking three times a week for 30 minutes nonstop will be completely under my control* and *I am sure that if I wanted to I could walk three times a week for 30 minutes nonstop*. Responses for these three items were recoded so that higher scores indicated greater control.

Finally, intentions were measured with three items, each with 4-response categories ranging from completely agree to completely disagree: *I intend to walk three times a week for 30 minutes nonstop*, *I am willing to walk three times a week for 30 minutes nonstop* and *I plan to walk three times a week for 30 minutes nonstop*. Responses were recoded so that higher scores indicated greater intentions to walk. Lastly, current behavior was measured with a dichotomous item: *Do you walk three times a week for 30 minutes nonstop*? Responses were yes or no.

### Analytical approach

The analysis, performed in 2015, followed a four-step process. First, we followed Fishbein and Ajzen’s recommendation [4] for identifying the most salient beliefs about walking three times a week for 30 minutes nonstop in this sample. Because the total number of beliefs may be greater than the number of participants, Fishbein and Ajzen [4] propose that the selection of salient beliefs can be guided by the following rule:

“[p]erhaps the most reasonable decision rule, and one that we would recommend, is to choose beliefs by their frequency of emission until we have accounted for a certain percentage, perhaps 75%, of all responses listed. For example, if the total number of responses provided by all participants in the elicitation sample was 600, a 75% decision rule would require that we select as many of the most frequently mentioned outcomes as needed to account for 450 responses” (p.103).

We completed this first step by hand and the following ones with Stata 11.

Second, we computed the means, standard deviations and Cronbach’s alpha coefficients for all the theory variables in order to assess the distributions and reliability of the measures. We set alpha levels of p < .05 to establish statistical significance. Third, we computed correlations among all the variables of the theory, and, finally, we conducted an ordinary least square model to regress intentions on its theoretical predictors, in order to measure the extent to which these individuals formed intentions based on attitudes, perceived social pressure and perceived behavioral control in regards to this behavior. Fishbein and Ajzen [4] recommend that, even at this initial step of the formative research, designers explore the relationships between intentions and its three cognitive antecedents, in order to identify the most relevant route for influencing behavior change. With that purpose, they recommend regressing intentions on attitudes, perceived social pressure and perceived behavioral control and then comparing the beta weights. This comparison, they suggest, would orient program practitioners to anticipate the cognitive construct carrying the greatest weight in the formation of intentions and, therefore, practitioners can target the intervention to that specific construct, by trying to influence its underlying salient beliefs.

### Ethics

Informed oral consent was obtained from all individual participants included in the study. This study received approval from the Institutional Review Boards of both Universidad Peruana Cayetano Heredia and Hospital Nacional Cayetano Heredia.

## Results

A total of 34 patients were interviewed, 58.8% (n = 20) were female. Their mean-age was 68.3 years (median 70; range 40–82) and 58.8% (n = 20) reported an education level of high school or more. Except for one participant with missing information, everyone was aware of their hypertension condition for an average of 8.4 years (median 5; range 0–30). About 41% (n = 14) of the patients were interviewed in the private clinic.

### Elicitation of salient beliefs

As shown in **Table 1**, patients perceived that among the most frequent consequences about their walking three times a week were *feeling better* or *it is good or bad for the body*. Further, patients perceived that the most common social referents that approve or disapprove of their walking three times a week were their *children*, *wife* and *grandchildren*; and among the most common social referents that walk and do not walk three times a week were their *children*, *neighbors*, *spouse* and *siblings*. Finally, patients perceived that among the most frequent facilitators and barriers that would allow or impede their walking three times a week were *(not) having someone to walk with*, *having work or responsibilities* or *having no time*.

**Table 1:** Salient beliefs about walking three times a week for 30 minutes nonstop.

	Advantages	Disadvantages
Behavioral beliefs	-Makes you feel better (relax, breath better, feel agile, improve health) -Good for your body (heart, bones, blood circulation, muscles) -Helps weight control -Exercise -Dr. recommends it	-Bad for your body (legs hurt, negatively impacts your health, agitation, hurt muscles, you can fall, get tired) -You go out alone -Have to deal with bad conditions (it can be hot, walk through building constructions, no traffic lights) -No time -Feel lazy
Injunctive normative beliefs	Approvers	Disapprovers
	-Children -Wife -Myself -Grandchildren	-Children
Descriptive normative beliefs	Doers	Non-doers
	-Children -Spouse -Grandchildren -Siblings -Neighbors -Daughter/son in law	-Neighbors -Children -Spouse -Siblings -Brother/sister in law
Control beliefs	Facilitators	Barriers
	-A park -Going shopping -Having someone to walk with -Feeling better (improving your health, relaxing) -Own motivation (willpower, like walking, interest and willingness) -Having to do something (work, chores at home, going for a walk)	-Work/responsibilities -Aches/illnesses -Not having someone to walk with -No time -Too hot, too cold or it rains -Indifference/apathy

### The constructs of the theory

The majority of participants (71%) reported engaging in the target behavior. Further, and though responses tended to be positive towards walking, all scales of the theoretical constructs showed good distributions and high internal consistency as measured by Cronbach’s alpha. The coefficient of internal consistency as well as the mean, standard deviation and minimum and maximum values for each of the theoretical constructs are shown in **Table 2**.

**Table 2:** Reasoned Action Approach constructs about walking three times a week for 30 minutes non-stop.

		Internal consistency	Mean	SD	Min-Max
Intention		.92	3.09	.42	2.00–4.00
Attitudes		.76	3.12	.26	2.63–3.63
Perceived social pressure		—	3.04	.37	2.50–4.00
	Injunctive norms	.90	3.03	.45	2.00–4.00
	Descriptive norms	.93	3.04	.43	2.00–4.00
Perceived behavioral control		.74	3.03	.33	2.34–4.00

Note: N = 34; Internal consistency was indexed with Cronbach’s alpha across all constructs, but with Pearson’s correlation for Descriptive norms. As suggested by Fishbein and Ajzen (4), perceived social pressure is not a scale but a combination of two scales—Injunctive and Descriptive norms—which correlated at *r =* .38.

Furthermore, the correlation between intentions and current behavior was positive and medium-sized (Spearman rho = 0.44, p<0.01). Intentions were independently and significantly associated with each of its three antecedents. **Table 3** shows the bivariate correlations among the variables of interest: intentions was associated with attitudes (Spearman *rho* = .42, p < .05), perceived social pressure (Spearman *rho* = .44, p < .05) and perceived behavioral control (Spearman *rho* = .56, p < .001). Finally, a multiple regression model (**Table 4**) showed that perceived behavioral control carried the greatest weight in the formation of intentions (β = 0.34, p < .10), yet none of the three estimates were statistically significant.

**Table 3:** Table of Spearman correlations among the Reasoned Action Approach constructs about walking three times a week for 30 minutes nonstop.

	Intentions	Attitudes	Perceived social pressure	Perceived behavioral control
Intentions	1.00			
Attitudes	.42*	1.00		
Perceived social pressure	.44*	.33	1.00	
Perceived behavioral control	.56***	.28	.44**	1.00

Note:N = 34

* p < .05

** p < .01

***p < .001

**Table 4:** Multiple regression of the Reasoned Action Approach constructs predicting intentions to walk three times a week for 30 minutes nonstop.

	B	Confidence intervals		SE (B)	*t*	Sig. (*p*)	β
Attitudes	.27	.-30	.83	.28	0.96	.34	.16
Perceived social pressure	.29	-.19	.76	.23	1.24	.22	.25
Perceived behavioral control	.43	-.05	.91	.23	1.84	.08	.34

Note: N = 34, Adjusted R^2^ = 0.34

## Discussion

Aiming to design an efficient intervention to promote physical activity among people living with hypertension, we examined the behavior, and its cognitive antecedents, of walking three times a week for 30 minutes nonstop among a sample of patients living with hypertension in the capital of Peru. Guided by the Reasoned Action Approach [4], one behavior change theory used extensively in health intervention design [9], we measured the extent to which the constructs proposed by the theory are associated with the selected behavior. We found that all measures of the theory constructs showed high internal consistency and good distributions and, for the most part, participants’ perceptions were positive towards walking three times a week for 30 minutes nonstop; in fact, the majority reported engaging in the behavior at the moment of the interview. Of interest, participants’ intentions to walk three times a week were significantly associated with self-reported behavior, and intentions were independently and significantly associated with each of its three cognitive antecedents.

As such, this study represents the initial step of a rigorous formative research that is essential to the design of a successful health intervention to motivate the initiation or maintenance of physical activity [9]. In low-resource settings, it is key to conduct theory-driven approaches to intervention development in order to avoid mistakes that can be costly in terms of money and time. Formative research is essential to any intervention design [22], particularly if human centered design approaches are to be considered.

After conducting a multiple regression model, the standardized beta coefficient with the greatest magnitude was that of perceived behavioral control. While all coefficients were non-significant, mainly due to the small sample size, it would seem that perceived behavioral control would be the construct carrying the greatest weight in the formation of intentions to walk, and, thus, it would be a candidate target for a health intervention promoting walking among people living with hypertension. A meta-analysis of studies examining physical activity, under the same theoretical approach, has found that attitudes and perceived behavioral control tend to carry the greatest weight in the formation of intentions to engage in physical activity [23]. Finding that perceived behavioral control is a main theoretical predictor of this behavior is relevant, because practitioners may develop an efficient intervention that would appeal to only that construct [4], as opposed to appealing to all constructs together with more complex interventions.

However, before engaging in any decision about what specific construct to target with a health intervention aiming to change behavior, Fishbein and Ajzen [4] recommend validating this initial conclusion with a second phase of formative research. In such phase, program designers can implement a survey with a larger sample to identify the salient beliefs that discriminate between intenders and non-intenders of the selected behavior [4]. In our study, we were capable of identifying those salient beliefs associated with walking three times a week for 30 minutes nonstop, but not to measure the extent to which these beliefs discriminated between intenders and non-intenders. Yet, in a subsequent phase, researchers could use the beliefs identified in this study to assess the correlations between each belief and a measure of intentions.

Of interest, our results revealed that among the salient behavioral beliefs were *feeling better* or *it is good or bad for the body*. The salient injunctive normative beliefs—that is, social referents that would approve or disapprove of one’s behavior—were *children*, *wife* and *grandchildren*. Among the salient descriptive normative beliefs—that is, social referents that engage or do not engage in the same behavior—were *children*, *neighbors*, *spouse* and *siblings*. Finally, the salient control beliefs about the facilitators and barriers of their behavior were, among others, *(not) having someone to walk with*, *having work or responsibilities* or *having no time*. These salient beliefs together can be further analyzed to identify an even smaller but meaningful selection of beliefs that can be targeted by a health intervention.

For example, based on the prior findings, the next step in this formative research would be to implement a study to measure individuals’ intentions to walk three times a week for 30 minutes nonstop as well as their perceptions about the beliefs outlined in this study. With such data, practitioners could examine the existing relationships among these variables and inform the development of message strategies. Recently, for example, Hennessy and his colleagues [6] measured parents’ beliefs associated with intentions to ban smoking in households in the United States, and found that while some salient behavioral and control beliefs discriminated between intenders and non-intenders, all the salient normative beliefs differentiated these two groups from each other. Such findings can help practitioners design message strategies that can be implemented with interventions promoting behavior change among a population of interest. In the case of hypertension, the study could find that the belief “*walking makes me feel better*” discriminates between intenders and non-intenders to walk, such that it would only be held by those who intend to walk but not by those who do not intend to walk; in this scenario, such belief would be selected for the next step in message development.

There is guidance in the health communication literature about how to plan message strategies based on studies using the Reasoned Action Approach [4, 24]. Hornik and Woolf [24] proposed three criteria to identify beliefs to be targeted by media interventions addressing health issues using formative research in the context of this theory: first, there should be a strong correlation between the measure of intentions and a selected belief; second, there should be enough individuals holding the opposite view on the selected belief, so that they can be moved into the right direction as a result of the intervention; and third, the selected belief has to be susceptible of change—that is, it cannot be a belief that is veridical or based on the direct experience of the individual, but rather it should be a belief that can be changed by an informational intervention [24]. Thus, those selected beliefs meeting the above criteria would be potential targets of health messages that promote, for example, physical activity among people living with hypertension.

Research in message design can further inform the selection of appeals or message formats that are most likely to influence those targeted beliefs [25]. A good example about how to construct messages is provided by Mendez and his colleagues [26], who designed and validated persuasive message appeals targeting attitudes to promote physical activity among people with coronary heart disease in Brazil. Such studies, including ours, contribute greatly to the design of more effective interventions that bridge the research-to-practice gap largely found in the public health arena [27], especially in guiding the development of patient-centered interventions.

One limitation of this study was the small sample size, thus calling for confirmation of our findings in a larger sample size. Also, the cross-sectional design of this study limits our ability to claim that perceived behavioral control is the main predictor of walking; it may well be that those who walk regularly feel more confident on their walking behavior, rather than the reverse. Future work can replicate this study with a longitudinal design. In addition, it is possible that the broad age range of the sample (from 40 to 82 years old) could hide differences in participants’ motivations to walk—in so far as younger adults may have different opportunities to walk than older ones; however, it should be noted that the question about barriers did elicit limitations for engaging in the study’s behavior that capture barriers for all age groups (e.g., work/responsibilities vs aches/illnesses, see Table 1). Furthermore, this study focused only on those individuals who have hypertension and know they have it; while implications of our results may not apply to other individuals outside this specific group, we suggest that similar communication strategies be used with those that have the condition but do not know they have it. Lastly, future formative research efforts could conduct a similar theory-driven approach with a focus on sedentary patients, so that beliefs that motivate walking could be identified among initiators. In our study, we did not discriminate patients who were current walkers from those who were non-walkers; that was a limitation as the literature indicates that habit is a predictor of physical activity [28].

## Conclusion

Overall and while cross-sectional in nature, our study provides elements to inform the design of an intervention that would motivate people living with hypertension to walk on a regular basis, irrespective of whether they are sedentary or current walkers. Though still preliminary, perceived behavioral control may be key to people’s decisions to walk on a regular basis; thus, a small set of control beliefs about barriers, including *(not) having someone to walk with*, *having work or responsibilities*, or *having no time*, could be targeted by a health communication campaign aiming to manage hypertension among individuals living with this condition.

## Supporting information

S1 File This is the S1_Qualitative dataset.docx. This is the dataset from the elicitation of salient beliefs.
(DOCX)

S2 File This is the S1_Quantitative dataset.dta. This is the dataset with demographics and constructs of the theory.
(DTA)

## References

[1] Fishbein M, Ajzen I. Belief, attitude, intention, and behavior: An introduction to theory and research Massachusetts: Reading; 1975.

[2] Ajzen I. The Theory of Planned Behavior. Organ Behav Hum [Internet]. 1991 12 [cited 15 sep 2016]; 50(2): 179–211. Retrieved from: https://doi.org/10.1016/0749-5978(91)90020-T

[3] Fishbein M. A Reasoned action approach to health promotion. Med Decis Making [Internet]. 2008 12 [cited 15 sep 2016]; 28(6): 834–844. Retrieved from: https://doi/10.1177/0272989X08326092 19015289 10.1177/0272989X08326092 PMC2603050

[4] Fishbein M, Ajzen I. Predicting and changing behavior: The reasoned action approach New York: Psychology Press; 2010.

[5] Riley W, Rivera D, Atienza A, Nilsen W, Allison S, Mermelstein R. Health behavior models in the age of mobile interventions: are our theories up to the task?. Transl Behav Med [Internet]. 2011 3 [cited 15 sep 2016]; 1(1): 53–71. Retrieved from: https://doi/10.1007/s13142-011-0021-7. 21796270 10.1007/s13142-011-0021-7 PMC3142960

[6] Hennessy M, Bleakley A, Mallya G, Romer D. Beliefs associated with intention to ban smoking in households with smokers. Nicotine Tob Res [Internet]. 2014a 1 [cited 15 sep 2016]; 16 (1): 69–77. Retrieved from: https://doi/10.1093/ntr/ntt119. 23943840 10.1093/ntr/ntt119

[7] Hennessy M, Bleakley A, Mallya G, Romer D. The effect of household smoking bans on household smoking. Am J Public Health [Internet]. 2014b 4 [cited 15 sep 2016]; 104(4): 721–727. Retrieved from: https://doi/10.2105/AJPH.2013.301634. 24524533 10.2105/AJPH.2013.301634 PMC4025695

[8] Chib A, van Velthoven MH, Car J. mHealth adoption in low-resource environments: a review of the use of mobile healthcare in developing countries J Health *Commun* *[Internet]* 2014 3 *[cited 15 sep 2016];* 20: 4–34. doi: 10.1080/10810730.2013.864735 24673171 10.1080/10810730.2013.864735

[9] Noar S. A 10-year retrospective of research in health mass media campaigns: Where do we go from here?. J Health Commun [Internet]. 2006 [cited 15 sep 2016]; 11(1): 21–42. Retrieved from: https://doi/10.1080/10810730500461059. 16546917 10.1080/10810730500461059

[10] Perel P, Casas J, Ortiz Z, Miranda J. Noncommunicable diseases and injuries in Latin America and the Caribbean: Time for action. Plos Med [Internet]. 2006 9 [cited 15 sep 2016]; 3(9): e344 Retrieved from: https://doi/10.1371/journal.pmed.0030344 16953660 10.1371/journal.pmed.0030344 PMC1570186

[11] Hernández-Hernández R, Silva H, Velasco M, Pellegrini F, Macchia A, Escobedo J, et al Hypertension in seven Latin American cities: The Cardiovascular Risk Factor Multiple Evaluation in Latin America (CARMELA) study. J Hypertens [Internet]. 2010 1 [cited 15 sep 2016]; 28: 24–34. Retrieved from: https://doi/10.1097/HJH.0b013e328332c353. 19809362 10.1097/HJH.0b013e328332c353

[12] Centers for Disease Control and Prevention. [Internet]. Atlanta: CDC 24/7; c2014. High Blood Pressure; [cited 15 sep 2016]. [1 screen]. Retrieved from: http://cdc.gov/bloodpressure/World Health Organization, 2013

[13] World Health Organization. A global brief on Hypertension. Silent killer, global public health crisis. [Internet]. 2013 [cited 15 sep 2016]. Retrieved from: http://apps.who.int/iris/bitstream/10665/79059/1/WHO_DCO_WHD_2013.2_eng.pdf

[14] Sánchez R, Ayala M, Baglivo H, Velázquez C, Burlando G, Kohlmann O, et al Latin American guidelines on hypertension. Latin American Expert Group. J Hypertens [Internet]. 2009 5 [cited 15 sep 2016]; 27: 905–922. Retrieved from: https://doi/10.1097/HJH.0b013e32832aa6d2. 19349909 10.1097/HJH.0b013e32832aa6d2

[15] Busse P. La desigualdad en la hipertensión Una investigación formativa en comunicación y salud. Lima: Instituto de Estudios Peruanos; 2014 55–89 p.

[16] Lee L, Watson M, Mulvaney C, Tsai C, Lo S. The effect of walking intervention on blood pressure control: A systematic review. Int J Nurs Stud [Internet]. 2010 12 [cited 15 sep 2016]; 47: 1545–1561. Retrieved from: https://doi/10.1016/j.ijnurstu.2010.08.008. 20863494 10.1016/j.ijnurstu.2010.08.008

[17] Albarracín D, Johnson B, Fishbein M, Muellerleile P. Theories of Reasoned Action and Planned Behavior as models of condom use: A meta-analysis. Psychol Bull [Internet]. 2001 1 [cited 15 sep 2016]; 127(1): 142–161. Retrieved from: https://www.ncbi.nlm.nih.gov/pmc/articles/PMC4780418/ 11271752 10.1037/0033-2909.127.1.142 PMC4780418

[18] Noar S, Palmgreen P, Chabot M, Dobransky N, Zimmerman R. A 10-year systematic review of HIV/AIDS mass communication campaigns: Have we made progress?. J Health Commun [Internet]. 2009 [cited 15 sep 2016]; 14(1): 15–42. Retrived from: https://doi/10.1080/10810730802592239. 19180369 10.1080/10810730802592239

[19] Jordan A., Piotrowski J., Bleakley A., & Mallya G. Developing media interventions to reduce household sugar-sweetened beverage consumption. Ann Am Acad Polit SS [Internet]. 2012 2 [cited 15 sep 2016]; 640(1): 118–135. Retrieved from: http://journals.sagepub.com/doi/abs/10.1177/0002716211425656

[20] Kallgren C., Reno R., Cialdini R. A Focus Theory of Normative Conduct: When Norms Do and Do not Affect Behavior. J Pers Soc Psychol [Internet]. 2000 10 [cited 15 sep 2016]; 26(8):1002–1012. Retrieved from: http://journals.sagepub.com/doi/abs/10.1177/01461672002610009

[21] Seclén-Palacín J, Jacoby E. Factores sociodemográficos y ambientales asociados con la actividad física deportiva en la población urbana del Perú. Rev Panam Salud Públ [Internet]. 2003 [cited 15 sep 2016]; 14(4): 255–264. Retrieved from: http://www.scielosp.org/pdf/rpsp/v14n4/18125.pdf 14662076

[22] Atkin CK. Theory and principles of media health campaigns 3^rd^ ed. California: SAGE Publications; 2001 49–68 p.

[23] Hagger M, Chatzisarantis N, Biddle S. A meta-analytic review of the theories of reasoned action and planned behavior in physical activity: Predictive validity and the contribution of additional variables. Jour Spo & Exe Psy [Intenret]. 2002 3 [cited 15 sep 2016]; 24(1):3–32. Retrieved from: https://doi.org/10.1123/jsep.24.1.3.

[24] Hornik R, Woolf K. Using cross-sectional surveys to plan message strategies. Soc Mar Q [Internet]. 1999 6 [cited 15 sep 2016]; 5(1): 34–41. Retrieved from: http://dx.doi.org/10.1060/15245004.1999.9961044.

[25] Cappella J. Integrating Message Effects and Behavior Change Theories: Organizing Comments and Unanswered Questions. J Commun [Internet]. 2006 8 [cited 15 sep 2016]; 56: S265–S279. Retrieved from: https://doi/10.1111/j.1460-2466.2006.00293.x

[26] Mendez R, Rodrigues R, Spana T, Cornélio M, Gallani M, Pérez-Nebra A. Validation of persuasive messages for the promotion of physical activity among people with coronary heart disease. Rev Lat-Am Enferm [Internet]. 2012 12 [cited 15 sep 2016]; 20(6): 1015–1023. Retrieved from: http://dx.doi.org/10.1590/S0104-11692012000600002 10.1590/s0104-11692012000600002 23258713

[27] Noar S, Harrington N, Helme D. The contributions of health communication research to campaign practice. Health Commun. 2010; 25: 593–594. doi: 10.1080/10410236.2010.496832 20845156 10.1080/10410236.2010.496832

[28] Rebar AL, Elavsky S, Maher JP, Doerksen SE, Conroy DE. Habits predict physical activity on days when intentions are weak. J Sport Exerc Psychol. 2014;36(2):157–65 doi: 10.1123/jsep.2013-0173 24686952 10.1123/jsep.2013-0173

