# Supplementary material for: Perceived behavioral control as a potential precursor of walking three times a week: Patient's perspectives
Source: PLoS One. 2018 Feb 16;13(2):e0192915. doi: 10.1371/journal.pone.0192915 (PMC5815616; doi:10.1371/journal.pone.0192915)
Supplement: S1 File — This is the dataset from the elicitation of salient beliefs. (DOCX) [file pone.0192915.s001.docx]

**Qualitative dataset**

**Principal investigator: Peter Busse, PhD**

**Numbers represent IDs of participants.**

**8. Tell me the advantages, the good or best things, of your walking three times a week for 30 minutes nonstop**

**01**

Te ayuda a los huesos, a la piel, al músculo, te hace sentir bien, te relajas.

**02**

Se siente más ágil, se desestresa.

**03**

Te oxigenas y previenes otras enfermedades de los huesos.

**04**

Mejora la salud física, da y haces actividad.

**05**

Bueno

**06**

Hacer ejercicio, todos los médicos recomiendan caminar.

**07**

Camina para estar un poquito mejor, es bueno

**08**

Para mantener los músculos, acción y moverse.

**09**

Se siente más liviana. Mucho mejor sería.

**10**

Sí es ventajosa porque a mi edad se tiende a subir de peso y ahora estoy recurriendo. Tonifica tus músculos. Me ha ayudado a bajar de peso.

**11**

Sí es bueno, por ejemplo por su enfermedad, hasta se puede hacer ejercicios, pero yo no lo hago.

**12**

Me encuentro bien caminando porque mi enfermedad no me molesta.

**13**

No hace mucho, ni ejercicios, pero allá sí hace más ejercicio. Si es que puede caminar normal, pero se agita mucho. (La van a operar de la vesícula).

**14**

Te relajas. No te sientes pesado, ves las cosas de la calle, no tienes preocupaciones por eso disfrutas, pero si caminas tenso, eso sí es malo.

**15**

Sería bueno porque bajaría de peso, a pesar de que no como antes, ahora no como en la noche, solo mi leche es lo que como por la noche.

**16**

Si caminas despacio, te relajas, respiras mejor, aquí a que tu corazón esté bien, te sientes liviana, tranquila.

**17**

Me ayuda a la circulación de la sangre, así no se me adormecen las piernas.

**18**

Da más energías, no suben ni bajas de peso.

**19**

Baja de peso, se lo han recomendado y la mejor manera es caminar “te ayuda a oxigenar el cuerpo”

**20**

Abriga el cuerpo, porque se siente calor, te ayuda a no tener dolor en los huesos

**21**

Que te ayudan a estar joven y en forma.

**22**

Mejora la salud

**23**

Que solo puedo salir con alguien, entonces vamos conversando, que es un ejercicio que me lo ha dicho el doctor y que tengo que hacer por mi salud y para controlar mi peso.

**24**

Te mareas un poquito caminando, pero en Lima no me gusta caminar mucho en la calle.

**25**

Será bueno para el corazón.

**26**

Respiras, conversas, ando como enamorado con mi esposa en la calle y nos vamos al parque o a las actividades de la municipalidad y nos distraemos.

**27**

Te relaja, despeja la mente, te ayuda a que estén bien los huesos.

**28**

Supongo que sí, porque el doctor recomienda que hay que tener actividad.

**29**

Uno se siente bien, te ayuda con el corazón.

**30**

Te ayuda a la circulación y a no tener várices.

**31**

Lo bueno es que ayuda, todo el cuerpo se llena de fuerza y puedes caminar, te ayuda a los huesos y no subir de peso, porque tengo que estar bien por mi corazón.

**32**

Me siento más ligera.

Ayuda a mi corazón a que trabaje menos y yo no me agito mucho.

**33**

Que es agradable, te hace sentir bien.

Ayuda a tu mente estar bien de salud y caminar, te relaja

**34**

Te ayuda a la circulación, ayuda a que tu cuerpo respire y que tu corazón trabaje bien.

**9. Tell me the disadvantages, the bad or worst things, of your walking three times a week for 30 minutes nonstop**

**01**

Te aburres en la casa si no caminas, pero si caminas y no sabes si te puedes caer o si hace calor te duele la cabeza.

**02**

A veces no hay tiempo.

**03**

A veces no hay tiempo o te parece que 30 minutos es mucho.

**04**

No hay desventajas

**05**

Está él dentro del trabajo.

**06**

No hay nada malo, es bueno.

**07**

A veces no te gusta o flojera

**08**

Adquirir de repente algún músculo que se entumezca.

**09**

Nada, siempre se tiene que caminar

**10**

No hay desventajas.

**11**

Un poco me fastidian los pies, los riñones no?

**12**

Si no me lo hago, me siento con dolores (refiere por ejercicios). No menciona desventajas por caminar. Comenta que hace terapia en cámara hiperbárica para disminuir dolores.

**13**

Ya no puede caminar pero allá en su pueblo sí pero se agita mucho; por eso su hijo no la deja.

**14**

Caminar tensa, preocupada de que te puedan robar, te agitas si vas muy rápido, te da calor, sudas, te pones roja, te puede dar un mareo.

**15**

Ahora por mi salud no puedo caminar, antes yo caminaba y me despejaba un poco.

**16**

Si vas rápido, te agitas, no sabes si tienes calor por el clima o son los bochornos, yo tengo problemas con el prolapso, entonces no puedo esforzarme mucho.

**17**

A veces si caminas muy rápido se te puede acalambrar o te puedes doblar el pie o te caes.

**18**

A veces si se camina con peso, te cansas.

**19**

No hay nada de malo, pero hay que caminar lento y con calma.

**20**

Sola no puedo ir, así que lo malo es no tener con quién ir.

**21**

Que no siempre se puede caminar porque a veces hay que ir en bus si te haces tarde.

**22**

Cuando tienes mucho física y te cansas.

**23**

Que me duelen las piernas.

**24**

A veces hay mucho ruido y carros y eso aturde un poco.

**25**

No tengo mucha costumbre de caminar, porque me movilizo en taxi o en mi auto.

**26**

A veces hay semáforos malogrados y no se puede caminar o no hay por dónde pasar por las obras de construcción.

**27**

Que cuando hace sol te puede dar sed (sonríe) o si caminas por el sol te agitas más.

**28**

Que se puede cansar y agitar

**29**

Recuerdo que caminaba con mi hermano y esposa y me siento triste.

**30**

Que a veces da un poco de pereza caminar o cansancio. También es que sola no camino y si salgo sola me preocupo si sabrán que salí.

**31**

A veces hay que caminar y no hay semáforos o estamos muy solos en la calle y puede ser peligroso.

**32**

Nada, Ahh! si llueve me resbalo un poquito.

**33**

Ahora no puedo mucho por el dolor, pero antes sí lo hacía.

**34**

Bueno, ahora por la faja (porque la usa luego de la caída) y rodillera duele un poco.

**10. Who are the most important people in your life who approve of your walking three times a week for 30 minutes nonstop? For example, your parents, siblings, partner, friends, etc.**

**01**

Yo creo que mis hijos

**02**

Mi esposa; yo mismo porque paro mucho tiempo sentado.

**03**

Mi esposa

**04**

Sí, me apoya mi esposo, sobre todo él.

**05**

Su preocupación y responsabilidad, camina sólo por trabajo.

**06**

Nadie

**07**

Missing

**08**

Nadie, yo sola

**09**

Hijos

**10**

Depende de mí.

**11**

No le han dicho, pero luego agrega que es bueno.

**12**

Mi esposa, mis hijos y especialmente Jesús

**13**

Sus hijos

**14**

Mi hija y su esposo me dicen “sal a relajarte”, te hace bien respirar aire puro.

**15**

No tengo con quién caminar, ya no salgo ni al mercado, generalmente va mi esposo. Si tuviese con quién ir para que me cuide si algo me pasa, sí iría.

**16**

Mi esposo, mis hijas, mi hermana.

**17**

Uno de mis hijos me recoge todos los días para caminar a eso de las 5pm. También mi hija aunque ya no sale porque ya va a dar a luz.

**18**

Mis hijos

**19**

Esposo e hijos

**20**

Mis hijos me dicen que salga a caminar

**21**

Mi pareja

**22**

Solo le apoya su esposa.

**23**

Mi hija y nieto

**24**

Mis hijos

**25**

Bueno, mi esposa me dice que camine.

**26**

Mis hijos

**27**

Mis hijos y nietos

**28**

La obligación por su salud, uno tiene que ser consciente. El trabajo. Lo haría por su cuenta, la familia se preocupa pero uno mismo lo hace.

**29**

Mis hijos

**30**

Todos me dicen tienes que caminar, el doctor te lo ha dicho.

**31**

Mi esposa, a veces camino con ella, mis hijos y nieto.

**32**

Mi hija y yerno

**33**

Mi familia

**34**

Mi esposa e hijos

**11. Who are the most important people in your life who disapprove of your walking three times a week for 30 minutes nonstop? For example, your parents, siblings, partner, friends, etc.**

**01**

No sé, si el médico lo dice, ellos (NOTA: *hijos*)van a querer.

**02**

Nadie

**03**

Nadie

**04**

No

**05**

Lo hace por trabajo.

**06**

Yo sola, no hay personas que me exijan

**07**

Sobrinas

**08**

Nadie

**09**

Nadie

**10**

No

**11**

No me han dicho: “tal día camina”

**12**

Mis dos hijos por trabajo

**13**

Sus hijos

**14**

Nadie

**15**

Tendría que ir acompañada pero ahora no puedo.

**16**

Nadie, pero a veces mi familia (esposo, hermana e hijas con quienes vivo), me dicen que no camine mucho.

**17**

Nadie

**18**

Nadie

**19**

Nadie

**20**

Cuando me dicen que no vaya sola (mis hijos)

**21**

Nadie

**22**

Su hija.

**23**

Nadie

**24**

Nadie

**25**

Mi hijo me dice que si no quiero, no lo haga.

**26**

Nadie

**27**

Missing data

**28**

La familia lejana, los primos, tíos

**29**

Nadie

**30**

Nadie

**31**

Nadie

**32**

Nadie

**33**

Nadie

**34**

Nadie

**12. Who are the people you know, or know of, who walk three times a week for 30 minutes nonstop? For example, your parents, siblings, partner, friends, etc.**

**01**

Mis hijos y nietos (aunque ellos corren)

**02**

Mi esposa generalmente camina más tiempo con todo lo que hace.

**03**

Mi esposa

**04**

Mi esposo

**05**

No lo sé.

**06**

Sí, mi hermana camina todas las mañanas.

**07**

Hermana, cuñado

**08**

Mis dos hijos

**09**

Hijos

**10**

Sí, algún vecino camina por el parque con los perros, haciendo sus aeróbicos, dando vueltas.

**11**

Sus hijitas para el trabajo.

**12**

No sé

**13**

Sus hijos

Allá (Nota de entrevistadora: En Cajamarca) no hay problemas que se agiten, como por mi enfermedad. Allá no se enferma.

**14**

Mi hija en España camina todo el día en el hospital, hasta mas de 30 minutos; mis vecinas.

**15**

Creo que a veces mi esposo camina, porque como es comerciante él hace compras, lo que yo a veces ayudaba y ahora no puedo.

**16**

Mis hijas, esposo, sobrinos

**17**

Mi hijo, mi nuera

**18**

Hijos, nuera

**19**

Esposo

**20**

Mis hijos, nietos

**21**

Mi esposa, hijos, amigas y amigos

**22**

Sus compañeros de trabajo.

**23**

Mi hija, nieto y las señoras de la parroquia.

**24**

Mis hijos

**25**

Mi hijo

**26**

Mis hijos y esposa (ella camina conmigo media hora de ida y regreso, 1 hora).

**27**

Hijos

**28**

Compañeros del trabajo

**29**

Mis hijos

**30**

Familiares, sobrina, esposo, hermano, nietos, hijos

**31**

Toda mi familia con la que vivo.

**32**

Vecinos, hija, yerno

**33**

Mis hijos y esposa y nieta

**34**

Mis hijos, mi esposa

**13. Who are the people you know, or know of, who do not walk three times a week for 30 minutes nonstop? For example, your parents, siblings, partner, friends, etc.**

**01**

No sé, creo que todos caminan.

**02**

Otros compañeros de transporte

**03**

Hermana, cuñado

**04**

Vecinos, hermanos, cuñados

**05**

No lo sé.

**06**

No lo sé.

**07**

Papa (por la edad), sobrina

**08**

En el sitio donde yo vivo todo el mundo camina.

**09**

Vecinos

**10**

Vecinos

**11**

Serán muy ancianitas o invaliditas.

**12**

No sé

**13**

Sus hijos

**14**

Mi esposo, por su trabajo

**15**

Mi hija porque se queda en casa con sus hijos.

**16**

Mis hermanas

**17**

Mi hija, porque ya no puede por su embarazo.

**18**

Vecinos, o muy viejitos o viejitos

**19**

A veces mis hijos

**20**

Algunos vecinos, vecinos viejitos

**21**

Nadie

**22**

Su hija, esposa, padre.

**23**

Mi vecina tampoco sale.

**24**

Nadie

**25**

Mi esposa

**26**

Vecinos

**27**

Algún vecino

**28**

Bueno, los congresistas, ellos no caminan, sólo están sentados.

Bueno, mi esposa va al mercado, camina en la cocina, tal vez camina más que yo, pero no creo que por media hora.

**29**

Nadie

**30**

Vecinos

**31**

Nadie

**32**

Nadie

**33**

Algunos vecinos y familiares

**34**

Algunos vecinos

**14.** **What makes easy, or what helps, your walking three times a week for 30 minutes nonstop? For example, a thing or a place, etc.**

**01**

Si es que cocinamos temprano, puedo dar una vueltita por unos 15 minutos y luego ir aumentando.

**02**

Apoyo a mi esposa en las compras de la casa para la semana pero sólo dos veces (fin de semana)

**03**

Interés en algo, comprar por ejemplo por el trabajo.

**04**

Pasear, salir por las ventas a comprar para el negocio.

**05**

Nada

**06**

Me ayudaría a bajar de peso y mejorar la salud.

**07**

Se siente más ágil y con ganas de hacer las cosas

**08**

Hacer las cosas de la casa, lleva a caminar, al mercado, al centro de Lima (compras)

**09**

“Su fuerza”, se dice “voy a caminar”

**10**

Su actividad de comercio de asumir un trabajo. Yo tengo un trabajo y por eso tengo que organizarme. Tendría que dejar de atender al público porque ella tiene un negocio.

**11**

El aire

**12**

La fuerza, la voluntad que tiene que poner. Si no tuviera el parque cerca igual caminaría.

**13**

Cuando me canso

**14**

Querer relajarme y salir, querer complementar mi dieta con ejercicio y caminar es salud, es un ejercicio para distraerte.

**15**

Muy fácil sería si no tuviera ese problema del soplo que dicen que es de nacimiento pero que ahora se ha agravado.

**16**

Cuando tengo tiempo y voy despacio si tengo que hacer compras o en casa.

**17**

Que mi hijo me recoja porque voy con él, si no puede ahí no salgo porque me da miedo que me vaya a casa.

**18**

Que tengo con quien salir, que me gusta caminar

**19**

Porque es para distraer la mente, una necesita respirar, si te quedas en casa te estresas. Yo salgo y camino hasta la plaza de armas, me siento un rato antes. Regresar y converso, luego me voy para mi casa con calma, te relaja.

**20**

Cuando tengo con quién salir

**21**

Que me gusta, me siento joven

**22**

Su trabajo, su salud

**23**

Si está mi hija o mi nieto, ellos me pueden acompañar, porque solo no puedo salir.

**24**

Mi hijo que pide que lo acompañe y de a poquitos me va llevando más y más lejos.

**25**

Por el momento nada.

**26**

Que voy acompañado con ella (señala a su esposa)

**27**

Que hago las cosas en la casa, salgo a comprar, que el parque está cerca.

**28**

Si me va a hacer bien yo camino, a parte la obligación de trabajo.

**29**

Mis hijos, hay un parquecito a unas 4 cuadras.

**30**

Que me apoyan en mi casa, el parquecito, ir con mi esposo

**31**

El querer estar bien y seguir viendo a mi familia, el que camino con mi señora y vecinos.

**32**

Que me gusta caminar

**33**

No puedo por ahora

**34**

Que camino para venir a terapia, que acompaño a mi esposa, caminamos por parque al ir y sobre todo después de recoger a nuestro nieto.

**15.** **What makes difficult, or what barriers are there to, your walking three times a week for 30 minutes nonstop? For example, a thing or a place, etc.**

**01**

No tengo con quién ir, porque yo puedo caminar, pero ellos me quieren acompañar y como no tienen tiempo, ya no salgo.

**02**

Mi trabajo porque soy chofer y más paro sentado.

**03**

Indiferencia en lo que tengas que hacer y lo postergas.

**04**

El tiempo, a veces falta.

**05**

Nada

**06**

Por mi esposo, tengo que alistarlo, preparar el desayuno, las obligaciones, cambiarlo.

**07**

Porque en la casa no queda nadie o puede llegar a llamar.

**08**

Ninguno (pero a veces desgano para hacer los queheceres)

**09**

A veces cuando siento un poco mal, dolor de las piernas, pies, débil de piernas

**10**

El negocio es esclavo: si salgo a caminar, pierdo ingreso. A veces tengo que programarme.

**11**

Mis riñones, hace un gesto de golpear el piso con el pie izquierdo.

**12**

Si no tuviera esa voluntad, estaría peor.

**13**

Su enfermedad

**14**

SI estoy muy ocupada en la casa o ese día desperté agitada, porque un día desperté acelerada, me latía el corazón y me sentía mareada.

**15**

El que me agito, me canso y si eso me pasa, me asusto y regreso a mi casa asustada, así salí un día y me regresé en taxi.

**16**

Cuando estoy con el dolor por el prolapso o si tengo que quedarme en casa.

**17**

Salir solo, o mejor dicho no tener con quien ir a caminar.

**18**

Cuando llovia, ya no salía mucho porque uno no puede caminar bien y se puede caer.

**19**

Nada, uno camina, paseando

**20**

Si es que nadie me puede acompañar.

**21**

Nada, todo es fácil y me gusta, porque después bailo.

**22**

Fala de tiempo.

**23**

Que si no tengo con quién salir, no podré salir a caminar.

**24**

Lo malo es el ruido o cuando no tengo con quién salir; prefiero quedarme en mi casa, pero el doctor me ha dicho que tengo que caminar.

**25**

Que me acostumbre a andar en auto.

**26**

Que si hace mucho calor o frío, luego nos da flojera salir.

**27**

Cuando hay muchas cosas que hacer en casa o estoy apurada, ya no camino sino tomo moto o combi.

**28**

El tiempo, para uno ocupado.

**29**

Cuando les toca exámenes o mucho trabajo ya no podemos salir.

**30**

Que a veces me da flojera, si hace frío ya no quiero salir.

**31**

Si hay mucho sol, o semáforos malogrados o arreglen el parque.

**32**

Nadie

**33**

El dolor en la pierna

**34**

Nada
